# Targeting epidermal growth factor‐overexpressing triple‐negative breast cancer by natural killer cells expressing a specific chimeric antigen receptor

**DOI:** 10.1111/cpr.12858

**Published:** 2020-06-27

**Authors:** Yan Liu, Yehui Zhou, Kuo‐Hsiang Huang, Xujie Fang, Ying Li, Feifei Wang, Li An, Qingfei Chen, Yunchao Zhang, Aihua Shi, Shuang Yu, Jingzhong Zhang

**Affiliations:** ^1^ The Key Laboratory of Bio‐Medical Diagnostics Suzhou Institute of Biomedical Engineering and Technology (SIBET) Chinese Academy of Sciences Suzhou China; ^2^ Changchun Institute of Optics Fine Mechanics and Physics Chinese Academy of Sciences Changchun China; ^3^ Soochow University Suzhou China; ^4^ Xuzhou Medical University Xuzhou China; ^5^ Tianjin Guokeyigong Science and Technology Development Company Limited Tianjin China; ^6^ Zhengzhou Institute of Engineering and Technology Affiliated with SIBET Zhengzhou China

**Keywords:** chimeric antigen receptor‐engineered natural killer cells, human epidermal growth factor receptor, triple‐negative breast cancer, xenograft mouse models

## Abstract

**Objectives:**

Traditional cancer therapy and regular immunotherapy are ineffective for treating triple‐negative breast cancer (TNBC) patients. Recently, chimeric antigen receptor‐engineered natural killer cells (CAR NK) have been applied to target several hormone receptors on different cancer cells to improve the efficacy of immunotherapy. Furthermore, epidermal growth factor receptor (EGFR) is a potential therapeutic target for TNBC. Here, we demonstrated that EGFR‐specific CAR NK cells (EGFR‐CAR NK cells) could be potentially used to treat patients with TNBC exhibiting enhanced EGFR expression.

**Materials and methods:**

We investigated the cytotoxic effects of EGFR‐CAR NK cells against TNBC cells in vitro and in vivo. The two types of EGFR‐CAR NK cells were generated by transducing lentiviral vectors containing DNA sequences encoding the single‐chain variable fragment (scFv) regions of the two anti‐EGFR antibodies. The cytotoxic and anti‐tumor effects of the two cell types were examined by performing cytokine release and cytotoxicity assays in vitro, and tumor growth assays in breast cancer cell line‐derived xenograft (CLDX) and patient‐derived xenograft (PDX) mouse models.

**Results:**

Both EGFR‐CAR NK cell types were activated by TNBC cells exhibiting upregulated EGFR expression and specifically triggered the lysis of the TNBC cells in vitro. Furthermore, the two EGFR‐CAR NK cell types inhibited CLDX and PDX tumors in mice.

**Conclusions:**

This study suggested that treatment with EGFR‐CAR NK cells could be a promising strategy for TNBC patients.

## 1 INTRODUCTION

The prevalence of triple‐negative breast cancer (TNBC) is approximately 15%‐20% among all patients diagnosed with the condition.^1^ TNBC tumors do not express estrogen receptor (ER), progesterone receptor (PR), and human epidermal growth factor receptor 2 (Her2). Hence, patients with TNBC cannot undergo regular immunotherapy or hormone therapy that targets these receptors. Patients with this form of breast cancer undergoing chemotherapy or radiotherapy experience several side effects.^2^, ^3^, ^4^ Furthermore, the recurrence rates of TNBC among patients who have undergone these therapies are higher than those among patients with other forms of breast cancer.^1^ Therefore, there is a need to develop a specific and effective therapeutic strategy to improve the outcomes of TNBC.

Studies that analyzed the epidermal growth factor receptor (EGFR)‐associated gene expression profile revealed that 45%‐70% of patients with TNBC exhibited EGFR overexpression, which was associated with poor prognosis.^5^, ^6^ Additionally, EGFR plays an important role in TNBC progression and EGFR mutation rarely occurs in patients with TNBC.^7^, ^8^, ^9^, ^10^, ^11^, ^12^, ^13^, ^14^, ^15^, ^16^ Furthermore, several anti‐EGFR monoclonal antibodies (mAbs) and small‐molecule tyrosine kinase inhibitors (TKIs) have been tested in clinical trials for the treatment of TNBC. However, the short‐term activity of these molecules limits their therapeutic efficacy.^5^, ^17^ Therefore, there is a need to develop an optimized EGFR‐targeted treatment for patients with TNBC.

Chimeric antigen receptor (CAR)‐engineered natural killer (NK) (CAR NK) cell therapy is one of the most promising immunotherapies for cancer.^18^, ^19^ Recently, third‐generation CARs were designed, which contain an extracellular binding domain, a hinge region, a transmembrane domain, and an intracellular domain. The extracellular binding domain includes a single‐chain variable fragment (scFv) derived from a tumor antigen‐reactive antibody. The intracellular domain includes both the signaling domain (CD3ζ), which mediates NK cell activation, and the co‐stimulatory domains (CD28 and 4‐1BB), which enhance the NK cell functions, such as proliferation, resistance to apoptosis, cytokine secretion, and persistence. Furthermore, CAR NK cell technology has been applied in several clinical or preclinical treatments for various tumors,^20^, ^21^ such as breast cancer,^22^ colorectal cancer,^23^ and glioblastoma.^24^

Chimeric antigen receptor‐engineered NK cell therapy has more advantages than the CAR‐engineered T‐cell (CAR T‐cell) therapy. Patients undergoing CAR NK cell treatment are unlikely to suffer from graft‐versus‐host disease (GVHD), which may occur in patients undergoing CAR T‐cell therapy.^25^, ^26^ The detrimental effects of CAR NK cells are much lower than those of CAR T cells, since activation of NK cells does not result in cytokine release syndrome as may be observed by the activation of T cells. Additionally, unlike CAR T cells, CAR NK cells can be generated from various sources, such as peripheral blood mononuclear cells (PBMCs), induced pluripotent stem cells, umbilical cord blood cells, human embryonic stem cells, and NK‐92 cell lines.^18^, ^27^, ^28^, ^29^

In the present study, EGFR‐specific CAR NK cells (EGFR‐CAR NK cells) were generated by fusing the scFv of an anti‐EGFR antibody to the artificially combined receptor molecules, in order to examine their anti‐tumor effects on TNBC cells. The anti‐EGFR scFv region recognized the wild‐type EGFR on TNBC cells. After recognition, the activated NK cells exerted cytotoxic effects on the TNBC cells exhibiting upregulated EGFR expression. Further, activation of the EGFR‐CAR NK cells significantly inhibited the progression of breast cancer in vitro and in vivo. The results of this study suggested that EGFR‐CAR NK cell immunotherapy could be the optimal treatment strategy for patients with TNBC in the future.

## 2 MATERIALS AND METHODS

### 2.1 Cell lines and culture

Human breast cancer cell lines (MDA‐MB‐231, MDA‐MB‐468, HS578T, and MCF7) were purchased from the American Type Culture Collection (ATCC). The cell lines were used for the experiments within 6 months. All the cell lines were cultured in Dulbecco's modified Eagle's medium (DMEM) (Gibco) supplemented with 10% heat‐inactivated fetal bovine serum (Gibco) and 1% penicillin‐streptomycin solution (Gibco) in a humidified incubator at 5% CO_2_ and 37°C.

### 2.2 Generation of EGFR‐CAR NK cells

The lentiviral vector containing third‐generation CAR (Con‐CAR) was purchased from iCARTab BioMed. The two DNA fragments of the anti‐EGFR scFv were cloned from the monoclonal hybridoma, which was previously established in the laboratory (Patent number: 20191158758.7).

The fused anti‐EGFR‐specific scFv (1 or 2) was cloned into the third‐generation CAR (Lenti‐EF1a‐scFv‐3rd‐CAR) with CD8 hinge, CD28 transmembrane domain, and intracellular signaling domains of 4‐1BB and CD3ζ. Con‐CAR was used as the control.

Peripheral blood mononuclear cells were isolated from the whole blood of healthy donors by Ficoll density gradient centrifugation. NK cells were obtained by stimulating the PBMCs in the NK cell‐specific medium (Dakewe) with 5% human serum (Sigma) for 14 days. The cells were incubated under saturated humidity conditions at 5% CO2 and 37°C. The 293T/17 cells were co‐transfected with CAR lentiviral plasmids along with pMD2.G and psPAX2 using the Lipofectamine 2000 reagent (Invitrogen). The lentiviral supernatant was collected after 2 days. Subsequently, the NK cells were transduced with lentiviral vectors carrying the DNA sequence that encoded the EGFR‐specific third‐generation CARs. The transfected cells were cultured under NK cell‐specific conditions for 3 days to obtain the EGFR‐CAR NK cells.

### 2.3 Real‐time polymerase chain reaction (PCR)

Total RNA was extracted using the RNeasy kit (OMEGA). The extracted RNA was subjected to reverse transcription using the PrimeScript RT reagent kit (Takara), following the manufacturer's instructions. The cDNA was subjected to real‐time PCR using SYBR Premix Ex Taq (Takara). The primer sequences used for real‐time PCR analysis are listed in Table 1.

**Table 1:** Primer sequences for real‐time PCR

Gene	Primer forward	Primer reverse
*EGFR*	AGTATTGATCGGGAGAGCC	CCAGGATAAATTGAATGGCAC
*CD3ζ*	GCCAGAACCAGCTCTATA	CCTCCGCCATCTTATCTT
*β‐actin*	AACCCTAAGGCCAACCGTGA	GTCTCCGGAGTCCATCACAA

### 2.4 Small interfering RNA (siRNA)

The EGFR‐targeting and negative control siRNAs were purchased from GenePharma (Suzhou). The siRNA sequences are listed in Table 2. The siRNA (50 mmol/L) was transfected into the cells using Lipofectamine RNAi MAX (Invitrogen).

**Table 2:** The sequence of short interfering RNA (siRNA)

Gene	Sense	Antisense
Negative control	UUCUUCGAACGUGUCACGUTT	ACGUGACACGUUCGGAGAATT
si‐EGFR	GAAUUAAGAGAAGCAACAUTT	AUGUUGCUUCUCUUAAUUCCU

### 2.5 Western blotting

The anti‐CD3ζ, anti‐EGFR, and anti‐β‐actin antibodies were purchased from Abcam, Cell Signal Technology, and Sigma, respectively. The anti‐rabbit and anti‐mouse secondary antibodies were purchased from Santa Cruz Biotechnology. The Western blotting analysis was performed according to the standard procedure.^30^, ^31^

### 2.6 Flow cytometry

The breast cancer cells and EGFR‐CAR NK cells were quantitated or isolated by flow cytometry using several fluorescence‐conjugated antibodies, following the manufacturer's instructions. The following reagents were used for this analysis: anti‐human CD3‐PE‐Cy7, anti‐human CD56‐PE, anti‐human CD69‐APC‐Cy7, mouse control PE, mouse control APC‐Cy7, mouse control PE‐Cy7, and Human TruStain FcX™ blocking solution purchased from BioLegend; anti‐EGFR antibody purchased from Cell Signal Technology; and goat anti‐rabbit IgG purchased from Abcam. The flow cytometric analyses were performed in a BD™ flow cytometer. The data were analyzed using FlowJo v10 software.

### 2.7 In vitro cytokine release assay

The human breast cancer cells (1 × 10^4^; HS578T, MDA‐MB‐468, MDA‐MB‐231, and MCF7) were co‐cultured with a suitable density of CAR NK cells in each well of the 96‐well flat‐bottom plates for 24 hours. The supernatant of the co‐culture was used to detect the levels of interferon (IFN)‐γ, granzyme B, and perforin (Dakewe) by enzyme‐linked immunosorbent assay (ELISA) (Dakewe).

### 2.8 Cytotoxicity assay

The human breast cancer cells (1 × 10^4^; HS578T, MDA‐MB‐468, MDA‐MB‐231, and MCF7) were co‐cultured with the optimized number of CAR NK cells in each well of the 96‐well flat‐bottom plates. The media containing dying and dead cells were collected for further analysis. The LDH cytotoxicity assay kit (Beyotime) and YOYO™‐3 Iodide (ThermoFisher) were used to measure the cytotoxic activity of the CAR NK cells, following the manufacturer's instructions. The cytotoxic activities were analyzed using an enzyme‐labeled instrument and a live cell imaging system.

### 2.9 Cell line‐derived xenograft (CLDX) mouse model

Female nude mice were purchased from Beijing Biocytogen Co.,Ltd and maintained under pathogen‐free conditions. The TNBC cells (5 × 10^6^ cells) were injected into the mammary fat pad of the female mice. NK cells (1 × 10^7^) were injected into the TNBC tumors on days 14, 21, 28, and 35. A Vernier caliper was used to measure the width and length of the tumors every week. The volume was calculated using the following formula: (1/2) × (*l*) × (*w*)^2^ [*l*, length; *w*, width].

### 2.10 Patient‐derived xenograft (PDX) mouse model

The medical ethics committee of the Suzhou Institute of Biomedical Engineering and Technology (A‐06) approved this method. The patients provided their signed authorization to use the human triple‐negative breast tumor tissues. The cells from these tissues were engrafted into the mammary fat pad of each mouse as described previously. NK cells (1 × 10^7^) were injected into the PDX tumors (at least >4 mm^3^) in mice at weeks 1, 2, and 3. The tumor size was measured as described previously.^32^

### 2.11 Immunohistochemical assay

Primary breast tumors and PDX tumors were fixed with 4% paraformaldehyde, embedded in paraffin blocks, and micro‐dissected into several thin sections. The sections were subjected to deparaffinization and antigen retrieval using citric acid buffer (pH 3.5) for 15 minutes. The specimens were incubated with 1% hydrogen peroxidase for 10 minutes. Subsequently, the specimens were incubated overnight with the horseradish peroxidase (HRP)‐conjugated antibodies against ER, PR, HER2, or EGFR (Cell Signaling Technology) at 4°C. The staining was performed using the HRP‐IHC kit, following the manufacturer's instructions.

## 3 RESULTS

### 3.1 Generation and characterization of EGFR‐CAR NK cells

**Figure 1:**
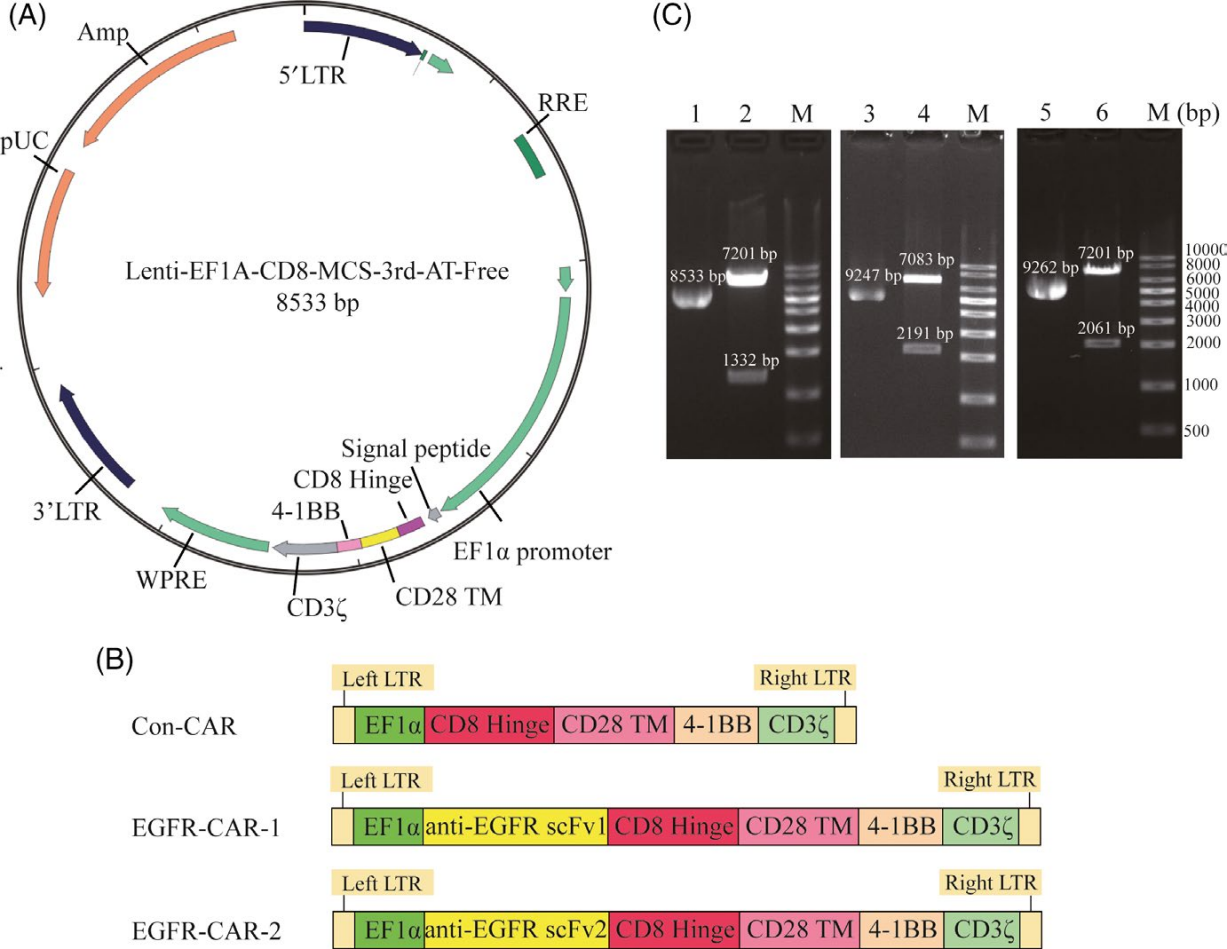
Construction of the chimeric antigen receptor (CAR) and gel electrophoresis of the plasmid and restriction enzyme‐treated DNA products. (A) Structure diagram of recombinant lentiviral vector containing the sequences encoding third‐generation CAR (Lenti‐EF1a‐scFv‐3rd‐CAR). (B) Schematic illustration of the lentiviral vector containing third‐generation CAR (Con‐CAR), epidermal growth factor receptor (EGFR)‐specific CAR‐1‐engineered (EGFR‐CAR‐1), and EGFR‐CAR‐2 constructs. (C) M: 1 kb DNA marker; Lane 1, untreated Con‐CAR plasmid; Lane 2, *N*coI‐treated Con‐CAR DNA products; Lane 3, untreated EGFR‐CAR‐1 plasmid; Lane 4, two *K*pnI‐treated EGFR‐CAR‐1 DNA products; Lane 5, untreated EGFR‐CAR‐2 plasmid; Lane 6, *N*coI‐treated EGFR‐CAR‐2 DNA products

**Figure 2:**
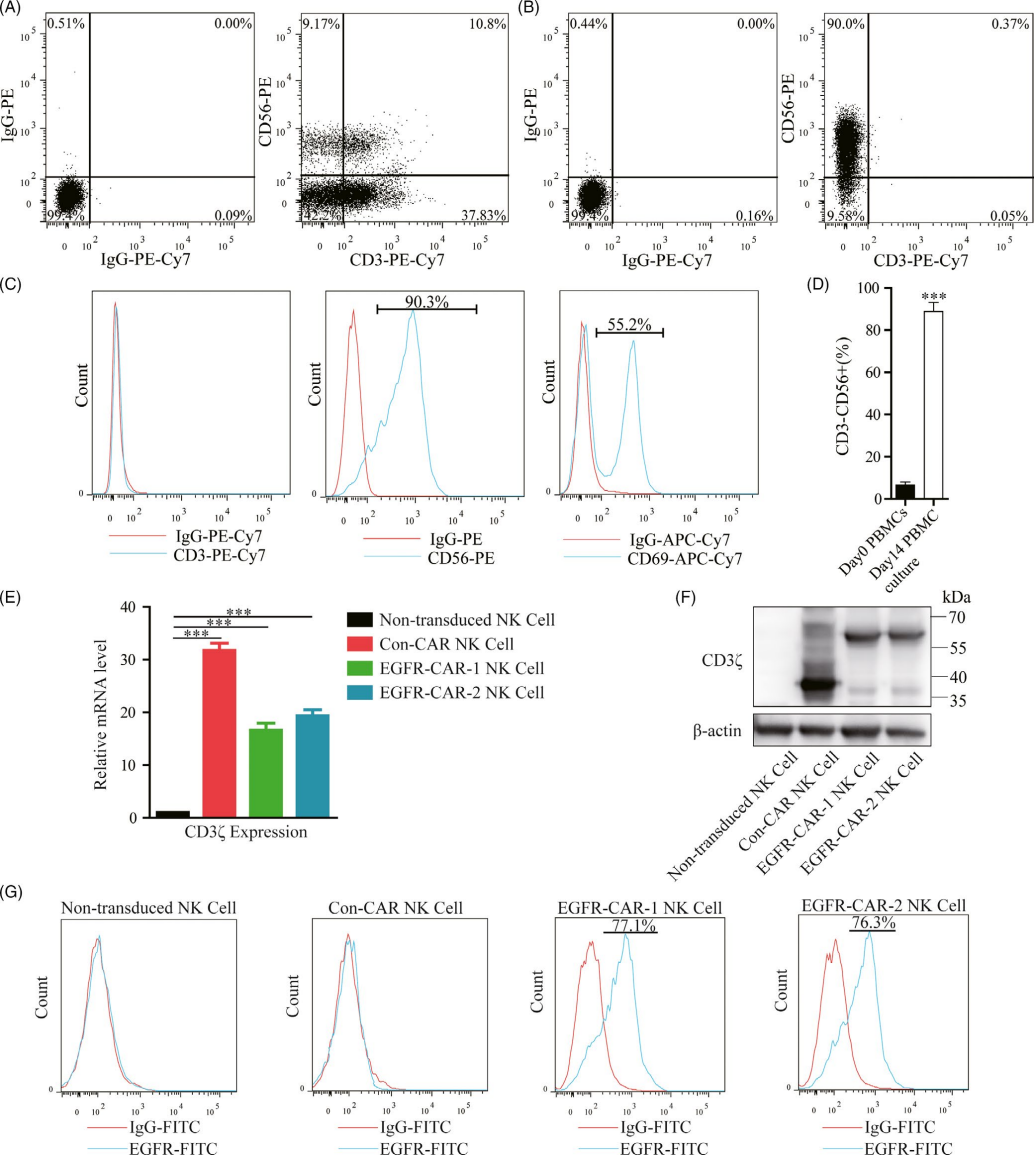
Generation, isolation, and characterization of epidermal growth factor receptor (EGFR)‐specific chimeric antigen receptor (CAR)‐engineered natural killer (NK) cells (EGFR‐CAR NK cells). (A) Flow cytometric analysis of phenotypic and subset composition of peripheral blood mononuclear cells (PBMCs) labeled with anti‐CD3‐PE‐Cy7, anti‐CD56‐PE. (B‐C) Flow cytometric analysis of phenotypic and subset composition of NK cells labeled with anti‐CD3‐PE‐Cy7, anti‐CD56‐PE, and anti‐CD69‐APC‐Cy7. (D) The percentage of CD3‐/CD56 + cells in day 0 PBMCs and day14 PBMC culture. (E) Real‐time PCR and (F) Western blotting analyses of the expression of exogenous CD3ζ in the non‐transduced NK cells, Con‐CAR NK cells, EGFR‐CAR‐1 NK cells, and EGFR‐CAR‐2 NK cells. β‐actin was used as an endogenous control. (G) The transduced NK cells stained with IgG‐FITC and EGFR‐FITC antibodies were detected by flow cytometry

The third‐generation EGFR‐CAR (Lenti‐EF1a‐scFv‐3rd‐CAR) was constructed by fusing the anti‐EGFR‐specific scFv (1 or 2) with the CD8 hinge, CD28 transmembrane domain, and intracellular signaling domains of 4‐1BB and CD3ζ (Figure 1A,B). The constructed lentiviral vectors carrying the CARs were verified by *N*coI/*K*pnI restriction digestion and gel electrophoresis (Figure 1C). Human primary NK cells were activated/isolated from the PBMCs cultured with (day14) or without (day0) NK cell‐specific medium. NK cells were characterized by flow cytometric analysis using the anti‐CD3, anti‐CD56, and anti‐CD69 antibodies. The percentage of the potential NK cells (CD3‐CD56+) isolated from the day 14 PBMC cultures was higher than the day 0 PBMCs (Figure 2A‐D). The potential NK cell population was further transduced with the lentiviral vectors that carried either of the two EGFR‐specific CARs (EGFR‐CAR‐1 and EGFR‐CAR‐2) or a third‐generation CAR as the control CAR (Con‐CAR). The generation of EGFR‐CAR or Con‐CAR NK cells was verified by real‐time PCR and Western blotting analyses using the primer of CD3ζ or anti‐CD3ζ antibody. The expression of CARs was analyzed in the transduced NK cells (Figure 2E,F). The non‐transduced or transduced NK cells treated with the EGFR‐ or IgG‐FITC were subjected to flow cytometry, to further characterize whether the EGFR‐CAR NK cells were able to recognize EGFR in vitro (Figure 2G). Approximately, 75% of the EGFR‐CAR‐1 or EGFR‐CAR‐2 NK cells were labeled with EGFR‐FITC (Figure 2G). Additionally, the transduction of CARs did not reduce the rate of proliferation of the NK cells (Figure S1). Thus, the generated EGFR‐CAR NK cells could specifically recognize EGFR in vitro.

### 3.2 EGFR‐CAR NK cells exert cytotoxic activity against TNBC cells by inducing cell lysis in vitro

The Western blotting analysis revealed that the protein expression levels of EGFR in the TNBC cell lines (HS578T, MDA‐MB‐468, and MDA‐MB‐231 cells) were higher than that in the non‐TNBC cell line (MCF7 cells) (Figure S2A). Additionally, the correlation between EGFR expression levels on the cell membranes and total EGFR levels in the four cell lines was examined by flow cytometric analysis using the anti‐EGFR antibody (Figure S2B‐E). The three TNBC cell lines and the MCF7 cell line were used to investigate the anti‐tumor activity of the EGFR‐CAR NK cells in this study.

**Figure 3:**
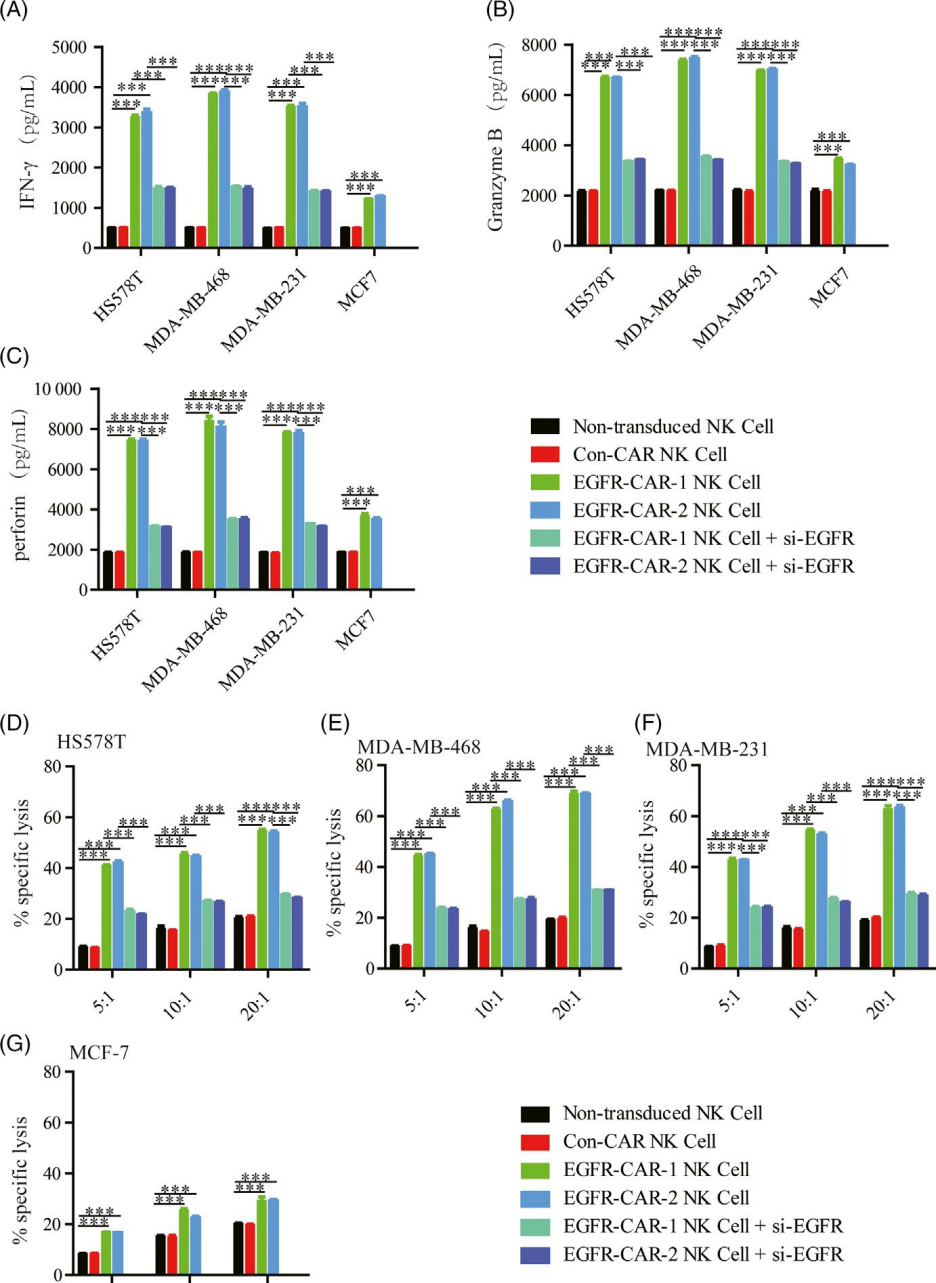
Cytokine release and cytotoxicity assay. Cytokine release of the effector non‐transduced natural killer (NK) cells, third‐generation chimeric antigen receptor (CAR)‐engineered NK cells (Con‐CAR NK cell), epidermal growth factor (EGFR)‐specific CAR‐1‐engineered NK cells (EGFR‐CAR‐1 NK cells), and EGFR‐CAR‐2 NK cells induced by the target cells. The effector cells were co‐cultured with the target cells (HS578T, MDA‐MB‐468, MDA‐MB‐231, and MCF7). The levels of (A) interferon (IFN)‐γ, (B) granzyme B and (C) perforin were analyzed in the supernatants of the co‐culture of the effector cells with the target cell at an E/T ratio of 10:1 for 24 h. Cytotoxicity of each group was measured by a standard lactate dehydrogenase (LDH) release assay. The effector cells were co‐cultured with the target cells (D) HS578T, (E) MDA‐MB‐468, (F) MDA‐MB‐231, and (G) MCF7 at an E/T ratio of 5:1, 10:1, 20:1 for 24 h. For each test, four duplicates were performed. The error bars represent the mean ± standard error of mean (SEM) of four biological replicates (n = 4). *t* test; ***P < .001

Cytokine release assays were performed to quantify the relative amounts of IFN‐γ, granzyme B, and perforin in the co‐cultured systems between NK cells (transduced or non‐transduced) and breast cancer cells (TNBC and non‐TNBC cells). The assays were performed to determine whether the EGFR‐CAR NK cells expressing either of the two EGFR‐CARs could be specifically activated by interacting with the TNBC cells (HS578T, MDA‐MB‐468, MDA‐MB‐231) exhibiting enhanced EGFR expression in vitro. The EGFR‐CAR NK cells co‐cultured with the TNBC cells exhibiting enhanced EGFR expression secreted significantly higher levels of IFN‐γ, granzyme B, and perforin than those co‐cultured with MCF7 (Figure 3A‐C and Table 3). Additionally, both the non‐transduced and Con‐CAR NK cells co‐cultured with either TNBC or non‐TNBC cells only secreted basal levels of IFN‐γ, granzyme B, and perforin (Figure 3A‐C and Table 3). Cytokine release assays were performed to quantify the cytokines secreted by the NK cells co‐cultured with the EGFR‐knockdown TNBC by siRNA in order to confirm the possible correlation between TNBC cells exhibiting enhanced EGFR expression and the activation of the EGFR‐CAR NK cells in vitro (Figure S3). Consistently, the lower EGFR expression of the TNBC cells led to lower cytokine secretion by the EGFR‐CAR NK cells (Figure 3A‐C and Table 3). These data suggested that activation of the EGFR‐CAR NK cells was likely induced by TNBC cells exhibiting upregulated EGFR expression in vitro.

**Table 3:** Cytokine release and cytotoxicity assay data

IFN‐γ	Non‐transduced T Cell	Con‐CAR‐T Cell	EGFR‐CAR‐1 T Cell	EGFR‐CAR‐2 T Cell	EGFR‐CAR‐1 T Cell + si‐EGFR	EGFR‐CAR‐2 T Cell + si‐EGFR
HS578T	488.12 ± 6.11	504.37 ± 2.95	3512.91 ± 66.11	3505.57 ± 105.7	1397.71 ± 77.65	1394.44 ± 61.91
MDA‐MB‐468	500.92 ± 6.27	502.36 ± 8.8	3829.35 ± 50.5	3873.12 ± 104.57	1515.99 ± 48.86	1456.33 ± 125.99
MDA‐MB‐231	496.95 ± 14.21	504.05 ± 12.31	3249.29 ± 92.03	3358.86 ± 100.09	1470.19 ± 118.44	1473.4 ± 60.12
MCF7	489.09 ± 15.08	495.61 ± 8.97	1220.01 ± 14.11	1274.07 ± 40.34		

Granzyme B	Non‐transduced T Cell	Con‐CAR‐T Cell	EGFR‐CAR‐1 T Cell	EGFR‐CAR‐2 T Cell	EGFR‐CAR‐1 T Cell + si‐EGFR	EGFR‐CAR‐2 T Cell + si‐EGFR
HS578T	2197.84 ± 40.37	2127.45 ± 100.69	6954.42 ± 61.32	6989.99 ± 115.86	3327.61 ± 59.6	3253.02 ± 41.83
MDA‐MB‐468	2197.38 ± 14.91	2169.75 ± 84.99	7359.36 ± 99.05	7452.03 ± 134.51	3530.96 ± 58.36	3392.68 ± 57
MDA‐MB‐231	2148.65 ± 86.49	2163.38 ± 42.97	6675.19 ± 107.28	6669.94 ± 71.3	3342.84 ± 52.79	3406.86 ± 40.66
MCF7	2158.03 ± 143.06	2140.04 ± 83.53	3410.83 ± 113.56	3199 ± 86.61		

Perforin	Non‐transduced T Cell	Con‐CAR‐T Cell	EGFR‐CAR‐1 T Cell	EGFR‐CAR‐2 T Cell	EGFR‐CAR‐1 T Cell + si‐EGFR	EGFR‐CAR‐2 T Cell + si‐EGFR
HS578T	1843.39 ± 31.02	1814.83 ± 50.21	7814.99 ± 81.96	7763.89 ± 250.35	3279.44 ± 18.37	3139.85 ± 51.5
MDA‐MB‐468	1834.99 ± 56.09	1856.42 ± 17.83	8391.36 ± 361	8087.56 ± 369.65	3482.25 ± 107.29	3478.75 ± 219.77
MDA‐MB‐231	1827.81 ± 51.7	1833.28 ± 54.35	7421.69 ± 147.89	7378.05 ± 169.2	3140.26 ± 47.38	3100.24 ± 27.19
MCF7	1859.73 ± 18.94	1861.13 ± 13.5	3669.41 ± 247.94	3486.72 ± 186.45		

E:T (HS578T)	Non‐transduced T Cell	Con‐CAR‐T Cell	EGFR‐CAR‐1 T Cell	EGFR‐CAR‐2 T Cell	EGFR‐CAR‐1 T Cell + si‐EGFR	EGFR‐CAR‐2 T Cell + si‐EGFR
5:1	8.79 ± 0.63	8.54 ± 0.47	40.97 ± 0.36	41.92 ± 1.29	22.92 ± 1.45	21.58 ± 0.55
10:1	15.93 ± 2.28	15.4 ± 0.28	45.13 ± 1.72	44.37 ± 0.84	26.91 ± 0.74	26.32 ± 1.04
20:1	20.15 ± 1.2	20.63 ± 0.68	54.53 ± 1.24	53.79 ± 1.12	29.3 ± 0.87	27.99 ± 0.65

E:T (MDA‐MB‐468)	Non‐transduced T Cell	Con‐CAR‐T Cell	EGFR‐CAR‐1 T Cell	EGFR‐CAR‐2 T Cell	EGFR‐CAR‐1 T Cell + si‐EGFR	EGFR‐CAR‐2 T Cell + si‐EGFR
5:1	8.66 ± 0.29	8.79 ± 0.52	44.23 ± 1.29	44.78 ± 1.04	23.47 ± 1.05	23.12 ± 0.53
10:1	15.8 ± 1.55	14.51 ± 0.34	62.46 ± 0.68	65.61 ± 1.23	27.13 ± 0.72	26.96 ± 1.73
20:1	19.11 ± 0.47	19.57 ± 0.66	68.58 ± 2.07	68.63 ± 0.72	30.71 ± 0.54	30.72 ± 0.53

E:T (MDA‐MB‐231)	Non‐transduced T Cell	Con‐CAR‐T Cell	EGFR‐CAR‐1 T Cell	EGFR‐CAR‐2 T Cell	EGFR‐CAR‐1 T Cell + si‐EGFR	EGFR‐CAR‐2 T Cell + si‐EGFR
5:1	8.49 ± 0.2	8.74 ± 1.07	42.53 ± 1.6	42.54 ± 0.63	23.68 ± 0.7	23.79 ± 1.03
10:1	15.76 ± 0.98	15.17 ± 0.78	54.24 ± 1.1	52.56 ± 1.57	27.32 ± 1	25.91 ± 0.83
20:1	18.77 ± 0.81	19.95 ± 0.73	62.72 ± 2.06	63.14 ± 1.6	29.01 ± 1.16	28.51 ± 1.23

E:T(MCF7)	Non‐transduced T Cell	Con‐CAR‐T Cell	EGFR‐CAR‐1 T Cell	EGFR‐CAR‐2 T Cell
5:1	8.37 ± 0.22	8.46 ± 0.28	16.77 ± 0.35	16.6 ± 0.14
10:1	15.27 ± 0.47	14.94 ± 1.38	25.42 ± 1.31	22.47 ± 1.21
20:1	20.21 ± 0.25	19.76 ± 0.35	29.2 ± 3.01	29.09 ± 1.03

Cytotoxicity assay was performed to quantify the specific lysis percentage by measuring lactate dehydrogenase (LDH) activity in the co‐cultured systems with the ratio between the effector (NK cells) and target cells (breast cancer cells) (E/T ratio). This assay was performed to investigate the ability of the activated EGFR‐CAR NK cells to specifically trigger TNBC cell death. As expected, higher E/T ratio between the EGFR‐CAR NK cells and the high‐EGFR‐expressing TNBC cells significantly elevated the percentage of the specific lysis in the co‐cultured systems (Figure 3D‐G and Table 3). Conversely, higher E/T ratio between the EGFR‐CAR NK cells and the low‐EGFR‐expressing MCF7 cells did not increase the percentage of the specific lysis in the co‐cultured systems (Figure 3D‐G and Table 3). In addition, higher E/T ratio between the EGFR‐CAR NK cells and EGFR‐knockdown TNBC cells did not increase in a manner similar to that between EGFR‐CAR NK cells and TNBC cells (Figure 3D‐G and Table 3). Furthermore, the results of the cell lysis assays (YOYO™‐3 Iodide staining) confirmed that the EGFR‐CAR NK cells triggered significantly greater lysis of TNBC cells exhibiting upregulated EGFR expression than the Con‐CAR NK or non‐transduced NK cells(Figure S4).

These data suggested that the activated EGFR‐CAR NK cells likely triggered cell lysis or death of the TNBC cells exhibiting upregulated EGFR expression in vitro.

### 3.3 Anti‐TNBC activity of the EGFR‐CAR NK cells in mouse models

**Figure 4:**
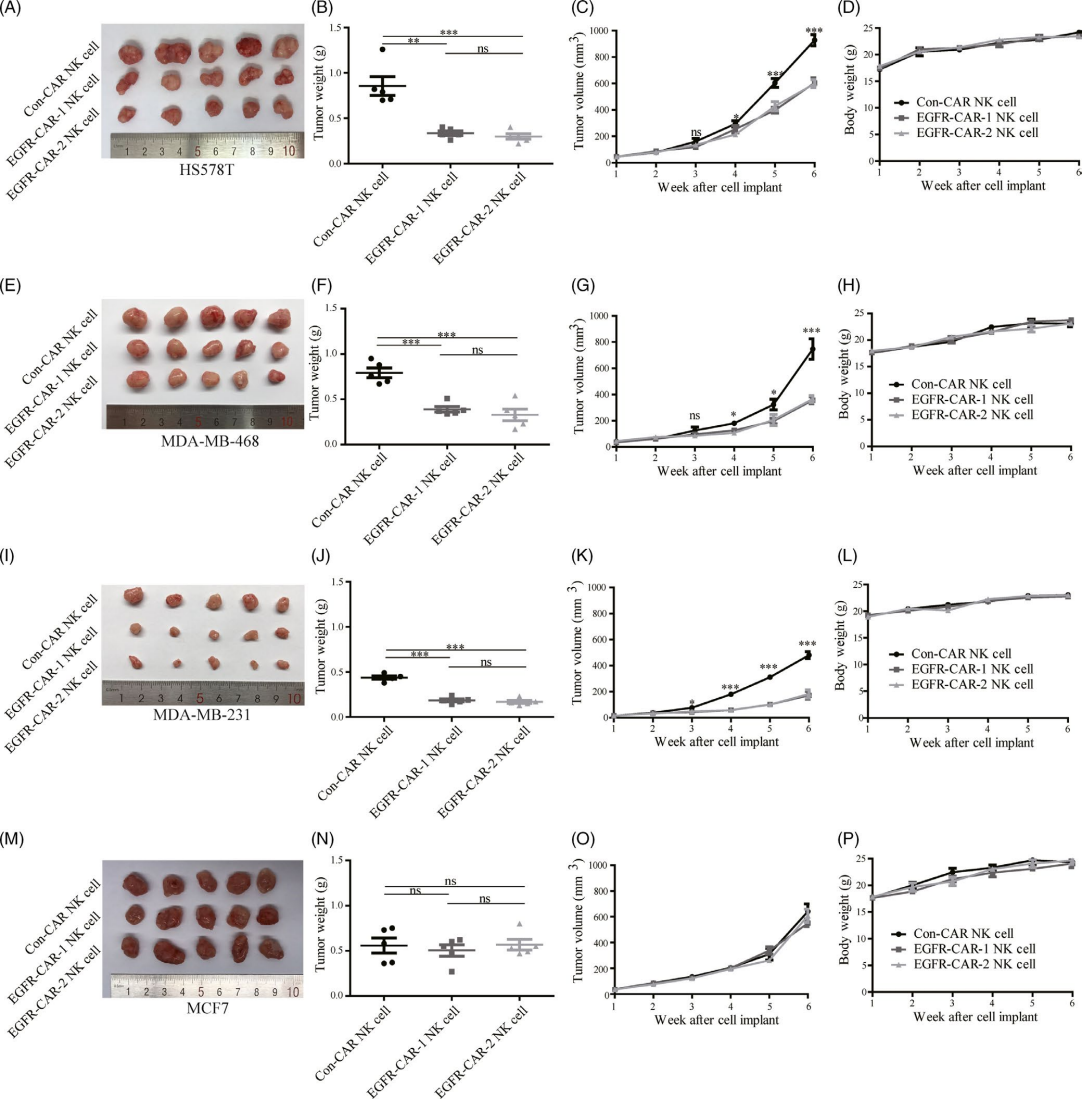
Epidermal growth factor (EGFR)‐specific chimeric antigen receptor (CAR)‐engineered natural killer (NK) cells (EGFR‐CAR NK cells) inhibited EGFR‐expressing triple‐negative breast cancer (TNBC) tumor growth in a xenograft mouse model. Compared to Con‐CAR NK cells, EGFR‐CAR‐1 NK cells and EGFR‐CAR‐2 NK cells decreased the tumor weight and tumor volume of (A, B, C) HS578, (E, F, G) MDA‐MB‐468, and (I, J, K) MDA‐MB‐231. However, treatment with EGFR‐CAR‐1 and EGFR‐CAR‐2 NK cells did not affect the tumor volume of (M, N, O) MCF7 and the body weight of the mice (D, H, L, P). The error bars represent the mean ± standard error of mean (SEM) (n = 5). *t* test; *P < .05; **P < .01; ***P < .001

**Figure 5:**
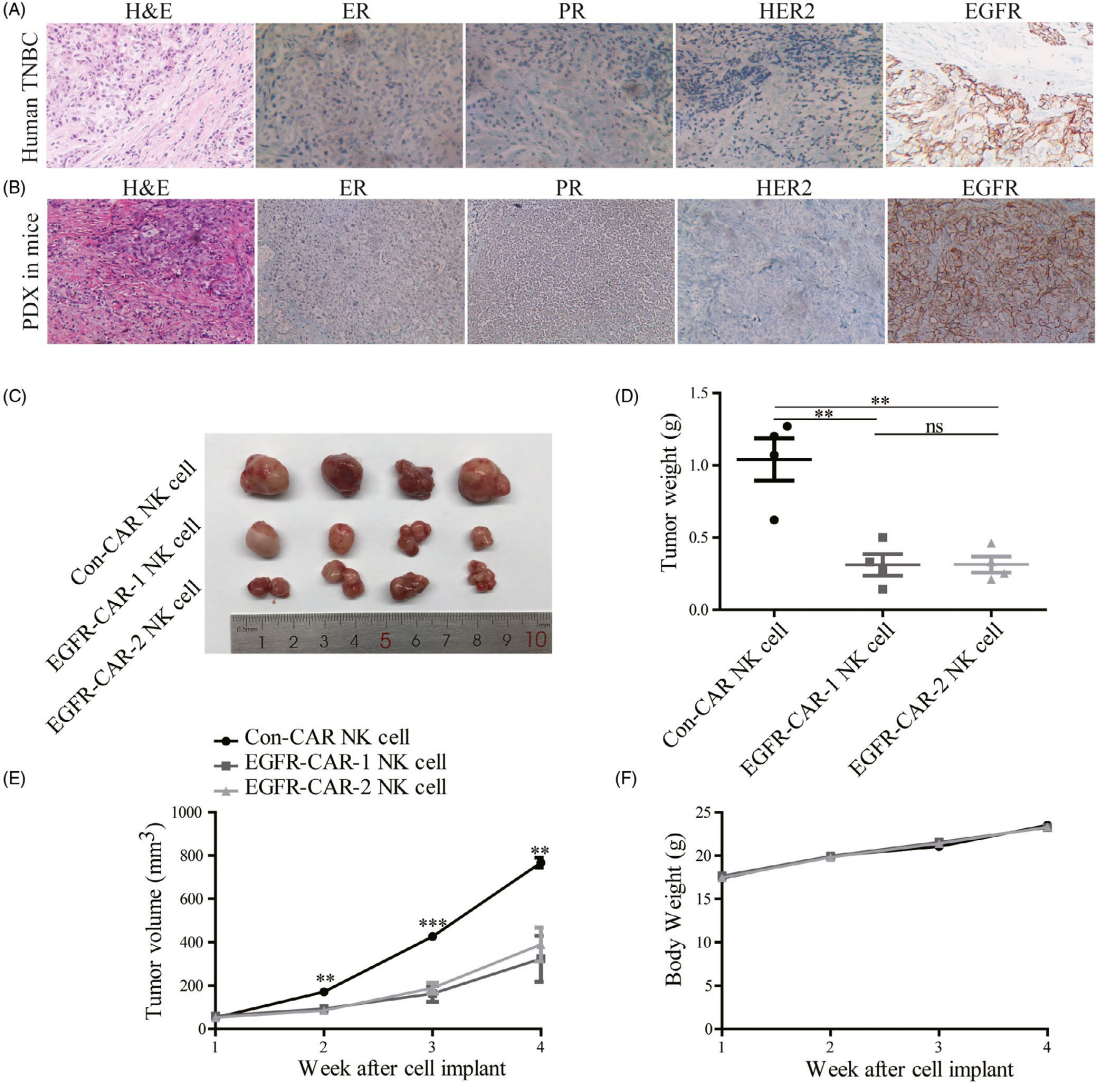
Epidermal growth factor receptor (EGFR)‐specific chimeric antigen receptor (CAR)‐engineered natural killer (NK) cells (EGFR‐CAR NK cells) inhibited the tumor growth of triple‐negative breast cancer (TNBC) exhibiting enhanced EGFR expression in the patient‐derived xenotransplant (PDX) mouse model. Immunohistochemical assay assessed estrogen receptor (ER), progesterone receptor (PR), human epidermal growth factor receptor 2 (HER2), and EGFR expression in (A) the clinical breast cancer sample and (B) breast tumors of NSG mice. Compared to Con‐CAR NK cells, the EGFR‐CAR‐1 NK cells and EGFR‐CAR‐2 NK cells decreased the (C, D) tumor weight and (E) tumor volume but did not affect the (F) body weight of mice. The error bars represent the mean ± standard error of mean (SEM) (n = 4). *t* test; **P < .05 and ***P < .01

Tumor growth assays were performed by inoculating the TNBC cell lines into the breast fat pad of the female nude mice that were injected with EGFR‐CAR NK cells or Con‐CAR NK cells on days 14, 21, 28, and 35 to assess the possible role of EGFR‐CAR NK cells in inhibition of CLDX TNBC tumor growth. The average weight and volume (size) of the xenograft TNBC tumors in the female nude mice treated with EGFR‐CAR‐1 or EGFR‐CAR‐2 NK cells were lower than those treated with the Con‐CAR NK cells (Figure 4A‐L). The MCF7 tumor growth could not be suppressed by the EGFR‐CAR NK cells (Figure 4M‐P). The average lifespan of TNBC tumor‐bearing mice treated with EGFR‐CAR NK cells or si‐EGFR was longer than that of the mice treated with Con‐CAR NK cells (Figure S5). Cells derived from TNBC patients were used to perform tumor growth assays to further investigate the role of EGFR‐CAR NK cells in the inhibition of PDX tumor growth. Consistently, the average sizes of the PDX tumors exhibiting upregulated EGFR expression in the female nude mice treated with EGFR‐CAR‐1 or EGFR‐CAR‐2 NK cells were also smaller than those treated with the Con‐CAR‐NK cells (Figure 5). Additionally, immunohistochemical analyses revealed that the protein (EGFR, ER, PR, and HER2) expression patterns of the PDX tumors in mice were similar to those of the original TNBC tissues from the patients (Figure 5A,B). Immunofluorescence imaging analysis revealed that CAR NK cells (CD56+) were present in the tumor core region after being injected into the tumor for 7 days (Figure S6). The body weights of the mice were not affected by treatment with EGFR‐CAR‐1, EGFR‐CAR‐2, or Con‐CAR NK cells in both xenograft models, suggesting that the tumor sizes were not affected by the health condition of the mice (Figure 4D,H,L,P and Figure 5F). These data indicated that the EGFR‐CAR NK cells likely inhibited the growth of TNBC tumors exhibiting upregulated EGFR expression in mice.

## 4 DISCUSSION

Chimeric antigen receptor‐engineere NK cells recognize their corresponding antigens via an antigen‐binding domain. CAR NK cells specifically recognize and kill tumor cells via antigen‐antibody binding. In this study, the EGFR‐CAR NK cells were generated by transducing a lentiviral vector containing the sequences encoding EGFR‐CARs (Figure 1). Western blotting and flow cytometry analyses indicated that the EGFR‐CAR NK cells could specifically recognize EGFR in vitro (Figures 2 and 3 and Figure S4). The activated EGFR‐CAR NK cells induced cell lysis or death of the TNBC cells exhibiting upregulated EGFR expression in vitro (Figure 3 and Figure S4). Furthermore, the EGFR‐CAR NK cells exerted a significant anti‐tumor effect on TNBC exhibiting upregulated EGFR expression in the two TNBC xenograft models (Figures 4 and 5). In addition, the tumor‐bearing mice treated with the EGFR‐CAR NK cells lived longer than the mice treated with Con‐CAR NK cells (Figure S5). Thus, our research indicated that EGFR‐CAR NK cells could be used for the development of a promising therapeutic strategy against TNBC exhibiting enhanced EGFR expression.

Epidermal growth factor receptor plays an important role in mediating cell proliferation, apoptosis, angiogenesis, and other cancer progression‐related functions.^33^, ^34^, ^35^, ^36^, ^37^ EGFR levels remain relatively high on the membranes of TNBC cells.^6^ Several EGFR‐specific mAbs and small‐molecule TKIs have been used in cancer therapy.^38^, ^39^, ^40^, ^41^, ^42^, ^43^ However, many patients with TNBC participating in trials responded poorly to these molecules. Additionally, the cancer cells in some patients with TNBC developed drug resistance during the trials. The development of immunotherapy has rendered CAR NK cell technology one of the most promising therapeutic strategies for solid cancers. The CAR NK cell technology has many advantages compared to the CAR T‐cell technology in targeted immunotherapy.^44^ For example, CAR NK cells do not cause GVHD. Furthermore, this immunotherapy does not cause cytokine release syndrome. Additionally, CAR NK cells can be generated from various sources.^25^, ^26^, ^27^, ^28^ In this study, EGFR‐CAR NK cells recognized EGFR more efficiently than the Con‐CAR NK cells (Figure 2G), and EGFR‐CAR NK cells were activated and secreted more IFN‐γ, granzyme B, and perforin when co‐cultured with TNBC cells exhibiting upregulated EGFR expression in vitro (Figure 3A‐C). Additionally, the activated EGFR‐CAR NK cells induced cytotoxic activity in TNBC cells exhibiting upregulated EGFR expression more dramatically than MCF7 cells in vitro, according to the data from both the LDH release and YOYO‐3 labeling assays (Figure 3 and Figure S4). These results suggested that cell lysis triggered by the EGFR‐CAR NK cells might be dependent on the amount of EGFR in breast cancer cells.

First‐generation antigen‐specific CAR NK cell immunotherapy was reported to be less effective against solid cancers than blood cancers.^45^ However, the third‐generation CAR NK cells that could mediate more intracellular signaling pathways demonstrated better anti‐tumor activity.^46^ The findings of this study revealed that EGFR‐CAR NK cells significantly inhibited TNBC exhibiting upregulated EGFR expression in the CLDX (Figure 4 and Figure S5) and PDX mouse (Figure 5) models.

The present study demonstrated that the activated EGFR‐CAR NK cells upregulated cytokine secretion, promoted cytotoxicity against the TNBC cells exhibiting upregulated EGFR expression in vitro, and inhibited tumor growth in mice without affecting mice bodyweight. However, EGFR is also expressed in several normal tissues.^47^, ^48^ Some EGFR‐specific immunotherapeutic trials involving cetuximab and nimotuzumab also reported side effects in patients.^49^, ^50^ Therefore, EGFR‐specific immunotherapy should be administered locally into the tumor rather than as systemic/intravenous injections. Moreover, the efficacy of EGFR‐CAR NK immunotherapy can be increased by simultaneously triggering TNBC cell apoptosis.^51^ Furthermore, CAR NK technology combined with mAbs or small‐molecule TKIs can improve the outcomes of breast cancer. This study provides a promising immunotherapeutic strategy for the treatment of patients with high‐EGFR‐ expressing TNBC.

## 5 CONCLUSION

In this study, we confirmed that EGFR‐CAR NK cells could effectively recognize TNBC cells exhibiting upregulated EGFR expression. Additionally, the two distinct EGFR‐CAR NK cells inhibited the growth of the TNBC tumor both in vitro and in vivo. Thus, EGFR‐CAR NK cells could be potentially applied in the treatment of patients with TNBC.

## CONFLICT OF INTERESTS

The authors declare no conflicts of interest.

## AUTHOR CONTRIBUTIONS

Study design: YL, SY, and JZ; Performed experiments and data analysis: YL, YZ, KH, YL, XF, LA, FW, QC, YZ, and AS; Characterization and optimization of lentiviral constructs and performing in vivo experiments: YL; Material support: YL and KH; Manuscript preparation: YL, YS, and JZ; Manuscript critical review: YL, YS, and JZ. All authors have read and approved the final manuscript.

## ETHICS APPROVAL AND CONSENT TO PARTICIPATE

Peripheral blood mononuclear cells were obtained from healthy donors and patients after informed consent based on protocols approved by the Suzhou Institute of Biomedical Engineering and Technology. The Suzhou Institute of Biomedical Engineering and Technology approved the study protocols. The mice were handled based on guidelines of the Institutional Animal Care and Use Committee of the Suzhou Institute of Biomedical Engineering and Technology (A‐06).

## Supporting information

Figure S1

Figure S2

Figure S3

Figure S4

Figure S5

Figure S6

## Data Availability

All data generated or analyzed during this study are included in the manuscript.
