# Supplementary figures and images for: Targeting epidermal growth factor‐overexpressing triple‐negative breast cancer by natural killer cells expressing a specific chimeric antigen receptor
Source: Cell Prolif. 2020 Jun 27;53(8):e12858. doi: 10.1111/cpr.12858 (PMC7445407; doi:10.1111/cpr.12858)

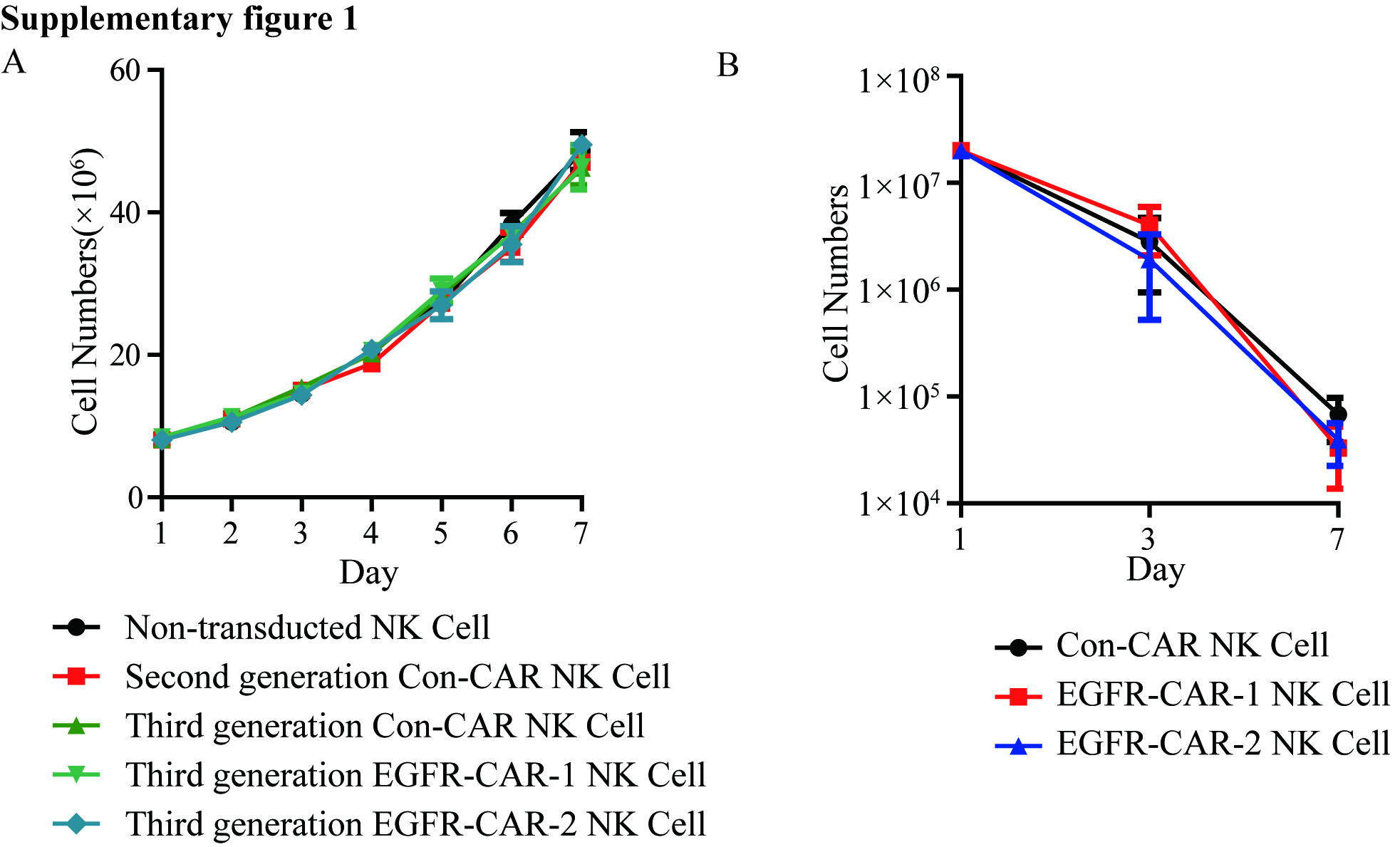

Supplement: Supplementary file 1 — Figure S1 [file CPR-53-e12858-s001.tif]

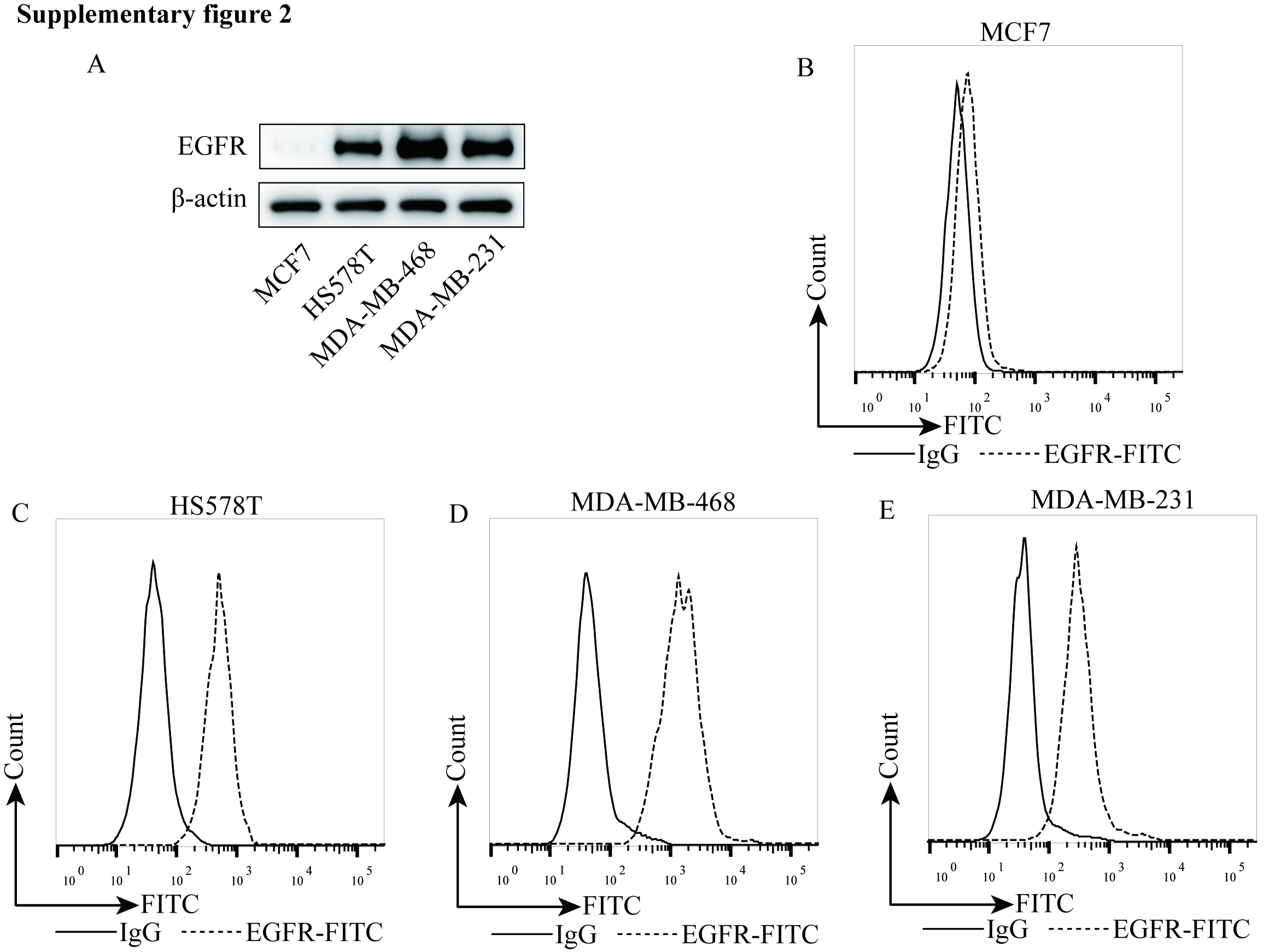

Supplement: Supplementary file 2 — Figure S2 [file CPR-53-e12858-s002.tif]

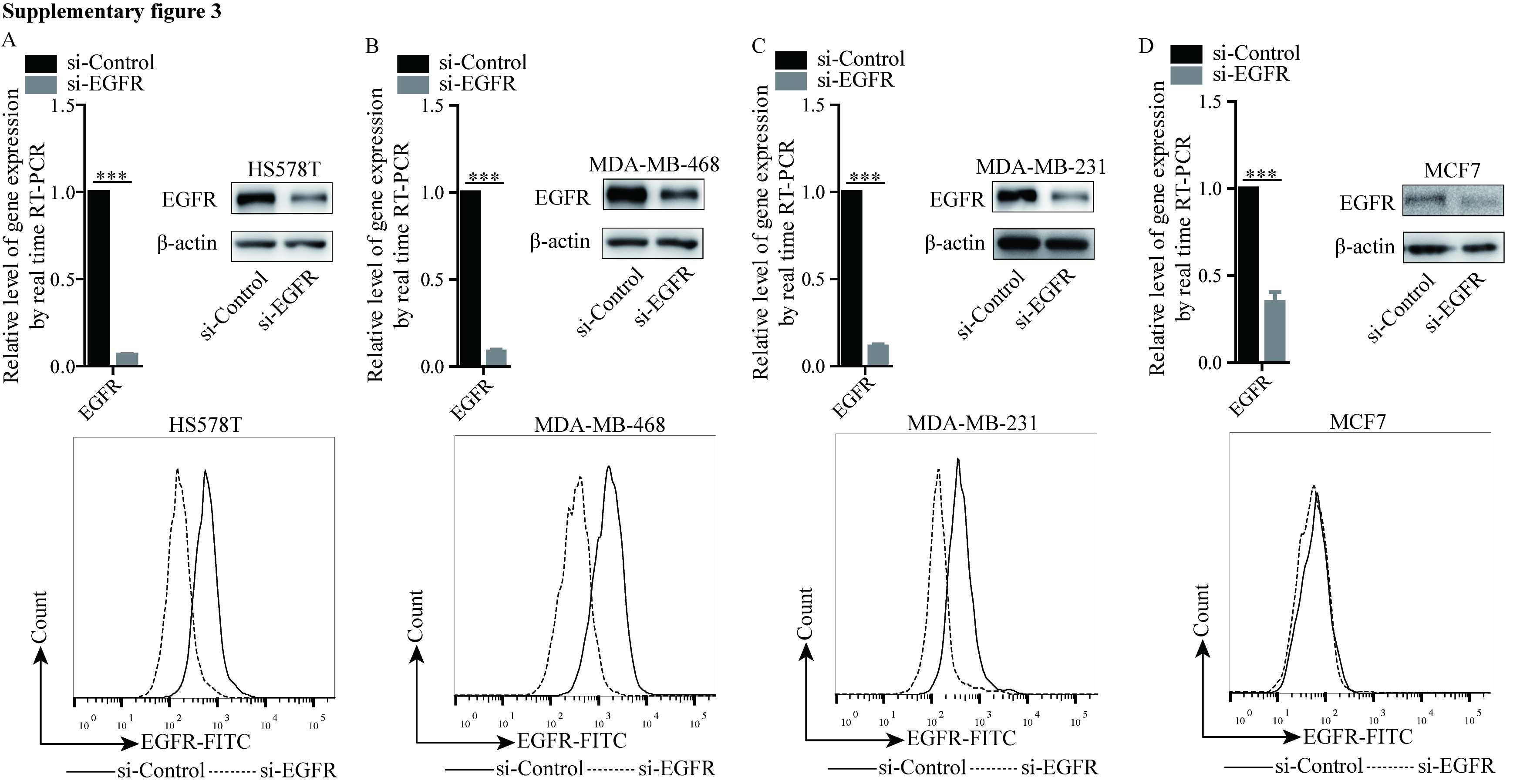

Supplement: Supplementary file 3 — Figure S3 [file CPR-53-e12858-s003.tif]

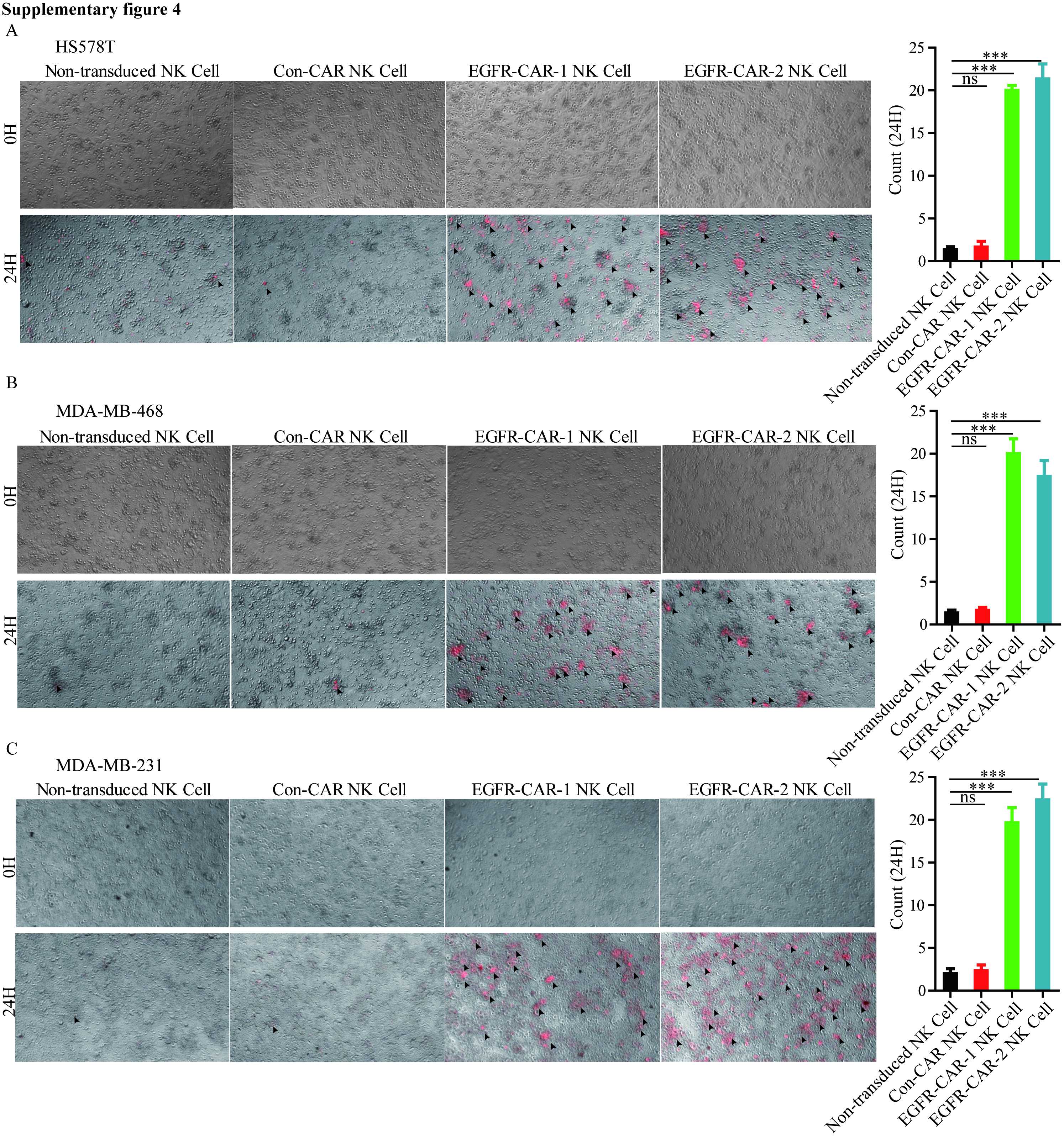

Supplement: Supplementary file 4 — Figure S4 [file CPR-53-e12858-s004.tif]

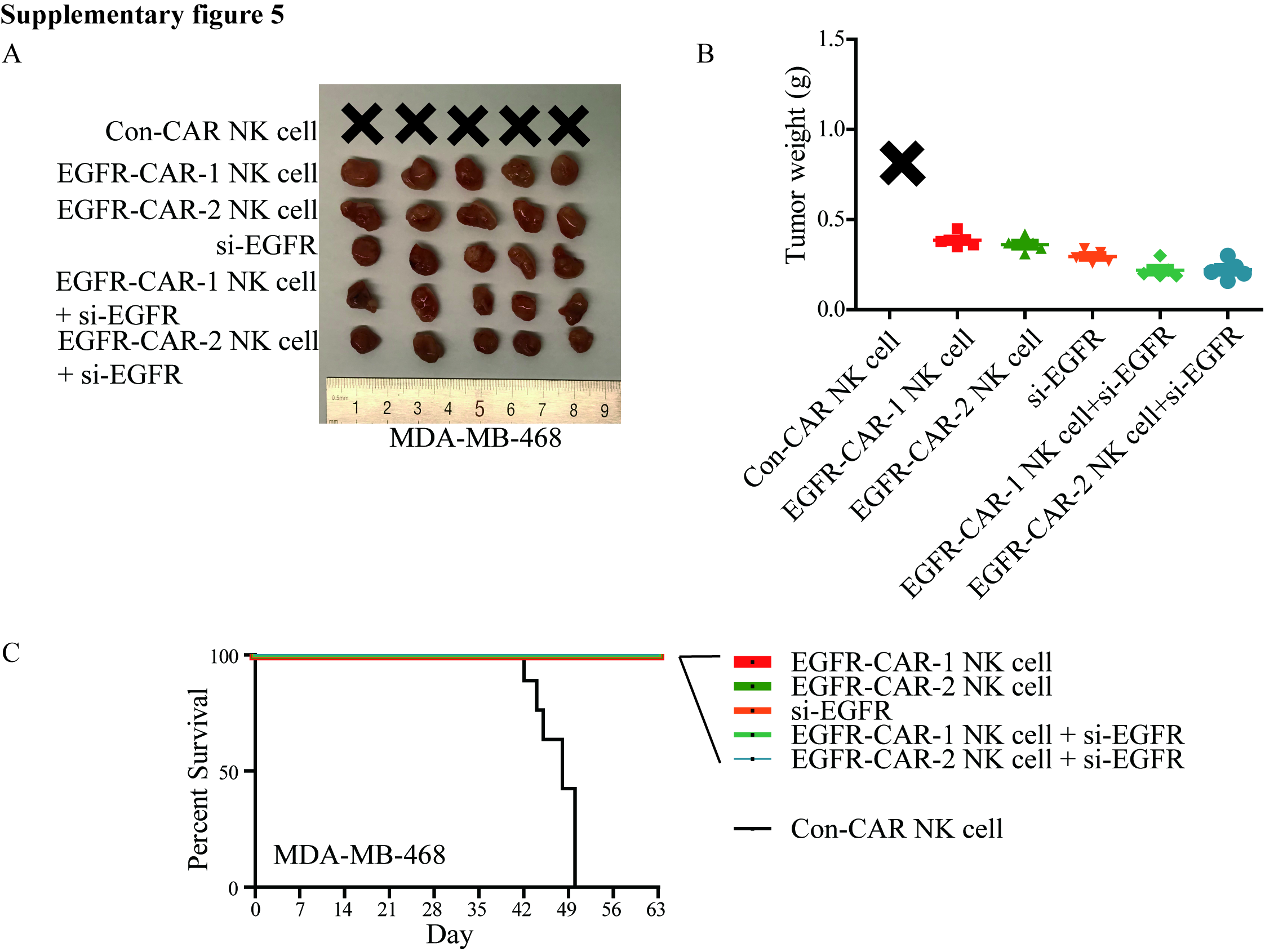

Supplement: Supplementary file 5 — Figure S5 [file CPR-53-e12858-s005.tif]

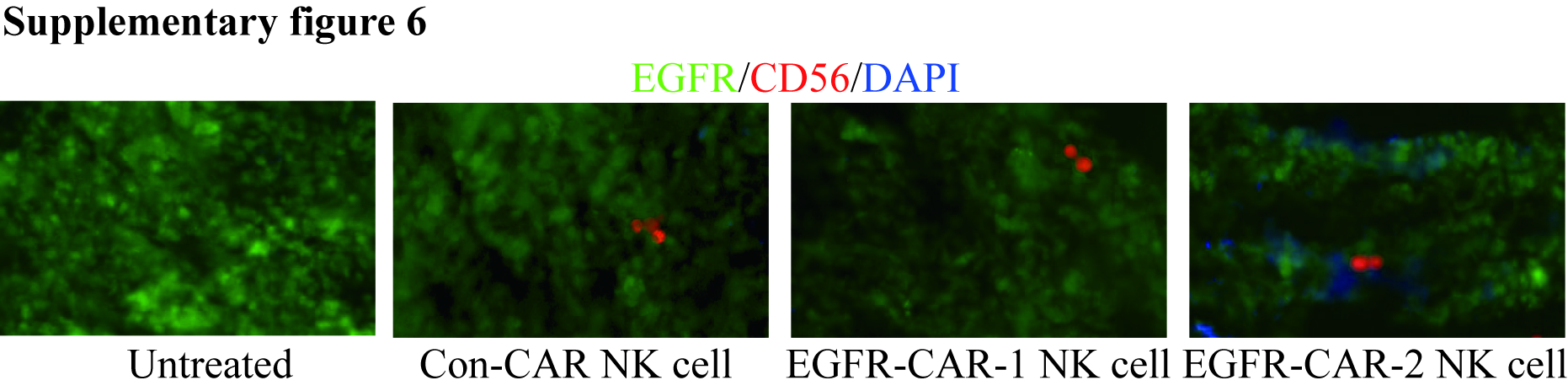

Supplement: Supplementary file 6 — Figure S6 [file CPR-53-e12858-s006.tif]
